# Estimation of Clustering Parameters Using Gaussian Process Regression

**DOI:** 10.1371/journal.pone.0111522

**Published:** 2014-11-10

**Authors:** Paul Rigby, Oscar Pizarro, Stefan B. Williams

**Affiliations:** 1 Australian Institute of Marine Science, Townsville, Queensland, Australia; 2 Australian Centre for Field Robotics, The University of Sydney, Sydney, New South Wales, Australia; Wake Forest University, United States of America

## Abstract

We propose a method for estimating the clustering parameters in a Neyman-Scott Poisson process using Gaussian process regression. It is assumed that the underlying process has been observed within a number of quadrats, and from this sparse information the distribution is modelled as a Gaussian process. The clustering parameters are then estimated numerically by fitting to the covariance structure of the model. It is shown that the proposed method is resilient to any sampling regime. The method is applied to simulated two-dimensional clustered populations and the results are compared to a related method from the literature.

## Introduction

In ecological studies, consideration of spatial structure can lead to useful insights regarding the process of interest (see, for example [1]–[3]). If the process exhibits clustering, then parameterised models which describe the spatial structure can provide a greater understanding of the behaviour of the species [4]. However, accurately determining parameters that describe a population can be a difficult problem within the natural environment. Measurements are often expensive and difficult to make, and so usually only a sparse sample of a population is available. In this paper we propose a new method for estimating the parameters of a cluster process from a sparse set of quadrat samples with arbitrary design, i.e. any sampling design such as transects, random etc. can be used without damaging the estimator.

The key theoretical framework upon which this work is built is the Gaussian process (GP), which can be defined as a collection of random variables, any finite number of which have a joint Gaussian distribution [5]. The Gaussian process framework can provide a useful tool for modelling stochastic processes and has seen much attention recently within the machine learning community, where it is used to solve regression and classification problems. GP regression has been widely studied within the field of geostatistics where it is known as kriging [6]. Under this guise it has also been given limited attention within the field of ecology (see, for example [7]–[9]). The kriging equations represent a special case of a Gaussian process.

In this paper we prove that the GP framework provides a useful route to estimating the parameters of a stochastic process, and has several advantages over previously published methods of achieving the same. The Neyman-Scott Poisson process is used as an example in this derivation, as this general cluster model has been widely studied and applied to naturally occurring populations (see, for example, [10]–[13]). The estimation is performed by fitting a theoretical covariance function to the empirical GP counterpart by numerical optimisation.

The standard approach to estimating the parameters of a cluster process is based upon the K-function developed by [14]. This estimation procedure assumes that the spatial process has been mapped over the whole survey area. This is not always practical and so alternative methods have been developed based upon line transect surveys. Most of these have only been developed to estimate the mean intensity of a population based upon a partial mapping, and cannot separate all of the parameters. However, Cowling [4] developed a method for estimating all the parameters in a two-dimensional Neyman-Scott process based upon a one-dimensional K-function along the transect line. An error in Cowling's derivation of the K-function was corrected in [15], where it was concluded that the corrected method clearly outperformed competing methods.

In this paper an alternative method for Neyman-Scott parameter estimation is developed, based upon GP regression. An experimental arrangement similar to Cowling is used, with the same transect sampling design. Experiments with alternative sampling designs are then tested to demonstrate the resilience of the technique.

## Methods

### Related Work

Within the ecological literature the standard approach to estimating the parameters of a cluster process based upon Ripley's K-function [14]. For a stationary isotropic process with intensity 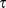, it is defined as 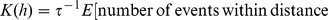


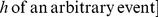
(1)


The K-function for a Neyman-Scott process of dimension 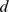 is given by Cressie [16]

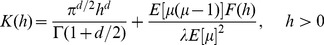
(2)where 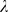 is the intensity of the parent process, 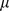 is the number of events per cluster and 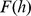 is the distribution function for the distance between two events in the same cluster.

If 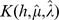 is the K-function evaluated at estimates of 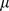 and 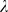, and 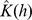 is a nonparametric estimator obtained from the data, then a least-squares estimate of 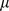 and 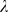 is obtained by minimising the *ad-hoc* criterion: 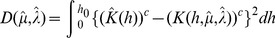
(3)where 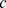 and 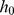 are tuning constants.

The above estimation procedure assumes that the spatial process has been mapped over the whole survey area. This is not always practical and so Cowling and Aldrin [4], [15] developed a method for estimating all the parameters in a two-dimensional Neyman-Scott process based upon a one-dimensional K-function along a transect line. The key steps in this method are reproduced below.

Cowling introduces a normal detection function 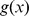, which is the probability of detecting an offspring at a distance 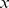 from the transect line. 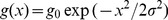
(4)


The two parameters 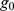 and 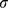 are typically estimated from external data, and are assumed known. The K-function for the detected points projected onto the transect line is then derived as 
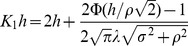
(5)where 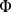 is the distribution function of the standard normal distribution. The empirical K-function is given by 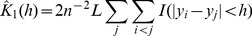
(6)where 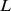 is the length of the transect, 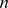 is the number of detected points, and 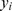 and 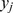 are the positions along the transect line. The parameters 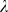 and 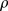 are then estimated by fitting the theoretical K-function to its empirical counterpart. Furthermore, given that 
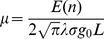
(7)



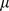
 can be estimated by substituting 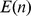 by the observed 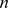, and 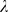 and 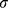 by their estimates.

### Gaussian Process Regression

In this section the GP regression methodology is outlined. For a fuller explanation the reader is directed to [5]. Consider a dataset 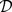


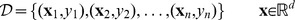
(8)which contains 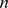 observations of some scalar variable 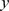, taken at locations 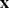. The dataset in equation 8 can be more compactly represented as 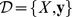 where 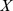 is an 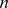 by 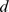 array of measurement locations, which will be referred to as *training points*, and 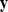 is a vector of the observations at those locations. Similarly, if predictions are to be made at more than one location, 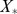 refers to an 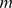 by 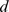 array of *test points*, and 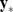 is the predicted output at these locations.

The distribution of the training outputs and the test outputs is jointly Gaussian with dimension 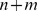, mean 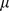 and covariance 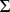


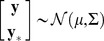
(9)

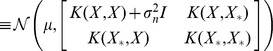
where 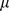 is a matrix containing the mean function evaluated at each of the 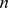 training points and 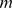 test points. 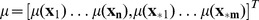
(10)


The covariance matrix in equation 9 has been partitioned to give the covariance matrices between the test points 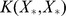, training points 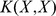, the cross covariance between both sets 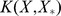 and its transpose 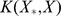. The values of 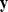 in the data set are not the actual function values, only noisy realisations of them. To account for this 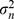 is added to the leading diagonal of the training covariance matrix.

Both the conditional and marginal distributions of a joint Gaussian distribution are Gaussian. It is this property which makes the Gaussian distribution appropriate for stochastic modelling, as closed form expressions for these distributions can be derived. Because 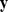 is known, it is possible to condition the joint Gaussian prior distribution on the observations [17] to give expressions for the mean and variance of the posterior GP:
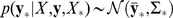
(11)where 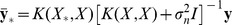
(12)

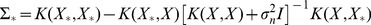
(13)


As in the case of kriging, the appropriateness of the GP model is entirely dependent on the choice of covariance function which has form arbitrarily selected by the user. Within GP literature [5] a popular form for the covariance function is the squared exponential 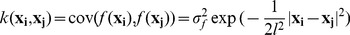
(14)where 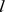 is a length scale that determines the strength of correlation between points. As two points are separated by a large difference, the covariance will tend to zero and the GP variance at test points far from measurements will tend to the underlying variance of the function 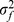. The parameters 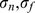 and 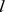 can all be varied, and doing so will affect the resulting GP model. The free parameters associated with any form of covariance function are referred to as *hyperparameters*. Also of interest is the *marginal likelihood* 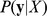 which can be obtained directly by considering that 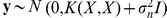. 
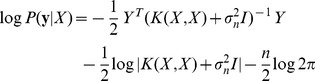
(15)


The marginal likelihood, or its logarithm, gives a measure of how well the covariance function explains the training data. The absolute value of the log marginal likelihood (LML) is dependent on the dataset but for any given dataset the LML can be used to compare different forms of covariance function and tune the hyperparameters.

Essentially, instead of trying to fit a parametrised model to the underlying function 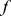, the GP (or the geostatistical) approach uses a parametrised model to describe the covariance. The key assumption is that the covariance of the process can be described using a simple parametric model.

As one would expect, in equation 12 the estimate of 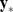 is a weighted average of the observations 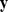. The form of equation 13 is also intuitive. The term 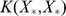 represents the prior covariance between the test points before any observations are made. When observations are made at locations 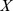 then the covariance in the prior will be decreased. The extent of this decrease depends upon the correlation between the observation locations and the test points, this is captured in 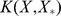. As a covariance function is by definition positive semi-definite, observations always result in information gain. However, these observations are also correlated with each other, and not as informative as would be expected if they were independent. Hence the inverse term 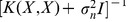 decreases the information gain accordingly. It is also interesting to note that 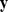 does not appear in equation 13, thus the variance only depends on the location of the observations, not on the value of the observations themselves.

The main computational burden in computing the mean function and the variance from equations 12 and 13 comes from the inversion of the training point covariance matrix, 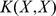.

### Estimation of Clustering Parameters

Consider a realisation of a Neyman-Scott process where invisible parent events are Poisson distributed with intensity 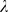 per unit area; each parent independently produces a Poisson distributed number of children with intensity 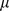; the positions of the children relative to their parents are independent and have an isotropic bivariate normal distribution with variance 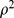 in the 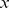 and 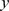 directions.

The survey field is of size 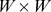 and is to be sampled using square quadrats of side 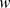, thus the measured quantity is the number of children observed in each quadrat. 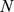 is the unknown number of clusters within the region 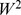, centred on parents 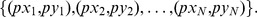



**Figure 1:**
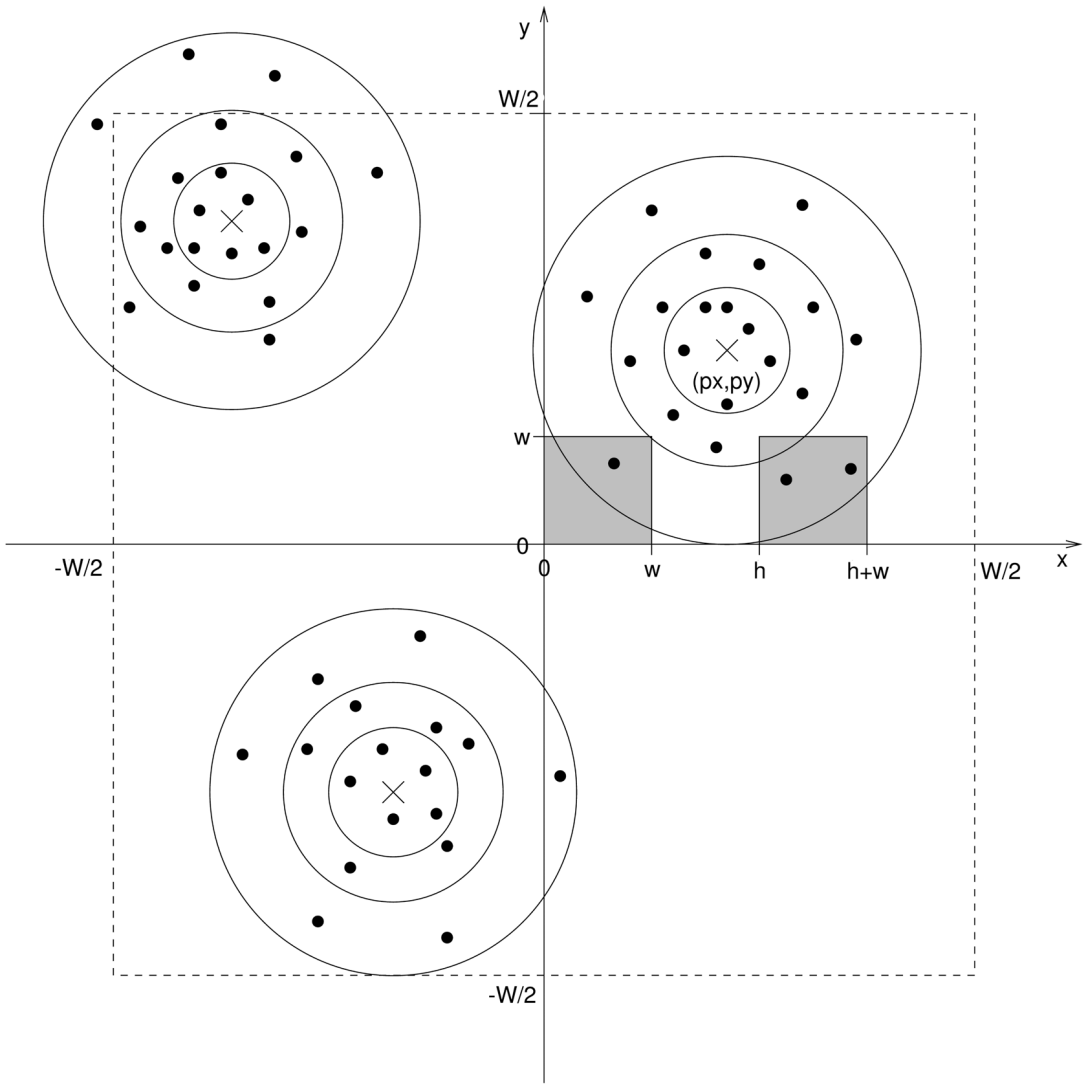
Two quadrats (shaded grey boxes) sampling a clustered distribution. Consider a realisation of a Neyman-Scott process where invisible parent events independently produces of children. The survey field is of size 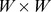 and is to be sampled using square quadrats of side 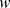, thus the measured quantity is the number of children observed in each quadrat.

The covariance of the counts 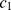 and 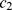 measured in two quadrats covering regions 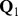 and 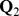, separated by distance 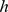 (see Figure 1) is defined as: 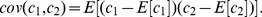
(16)


Let 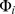 be a bivariate Gaussian distribution centred at a parent location 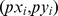


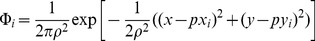
(17)then the expected number of children from cluster 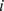 that will fall within 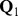 is given by 
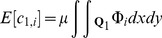
(18)and the covariance between two quadrats separated by distance 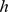 can be obtained by substituting into equation 16: 
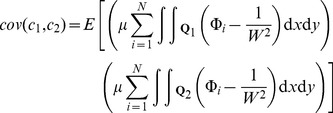
(19)


Expanding this expression gives 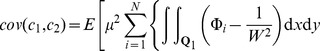


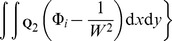


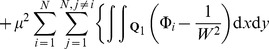


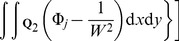
(20)which because of the independence of the cluster locations can be simplified to 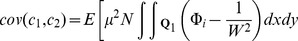


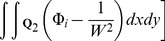
(21)


The placement of cluster 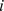 is arbitrary, and could be anywhere within the region 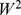 with equal probability. Evaluating the expectation operator, and noting that 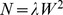, gives 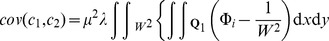


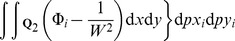
(22)


In this implementation Equation 22 was evaluated by solving the inner integrals 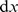 and 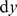 to give a closed form solution in terms of the error function, and then the outer integrals 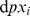 and 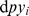 were evaluated numerically.

## Results and Discussion

### Estimation of simulated process parameters

**Figure 2:**
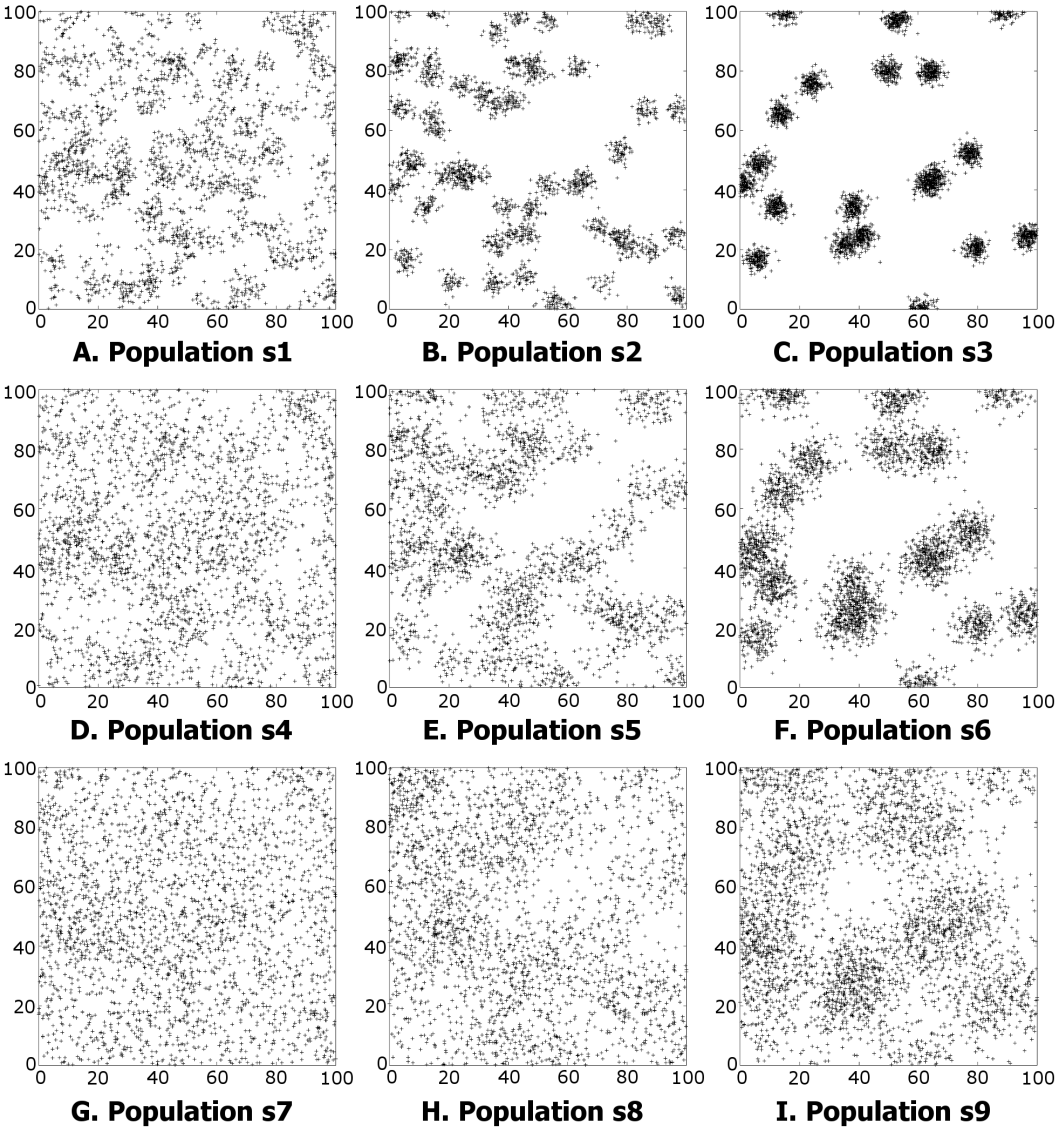
One realisation of Neyman-Scott processes for populations s1…s9. The Neyman-Scott parameters used to generate each population are given in Table 1. 100 realisations of each population were randomly generated and used to test the GP estimator.

Events were simulated from nine clustered populations using a two-dimensional Neyman-Scott model within a 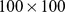 square survey area. In each case the product 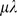 was equal to 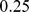, hence the expected number of events is the same for each population, however the extent of clustering varies. One realisation of each population is shown in Figure 2 and the simulation parameters are given in Table 1. Note that s3 exhibits the most clustering, and s7 the least. Edge effects were minimised by simulating the process for a 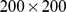 region, and then only considering events lying within a central 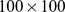 square.

**Table 1:** Population parameters.

Population	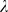	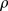	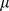
s1	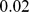	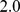	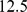
s2	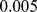	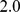	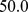
s3	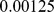	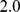	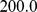
s4	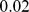	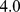	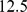
s5	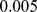	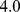	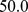
s6	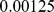	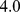	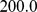
s7	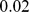	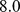	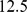
s8	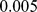	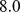	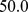
s9	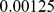	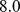	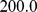

Parameters of simulated clustered populations.

**Figure 3:**
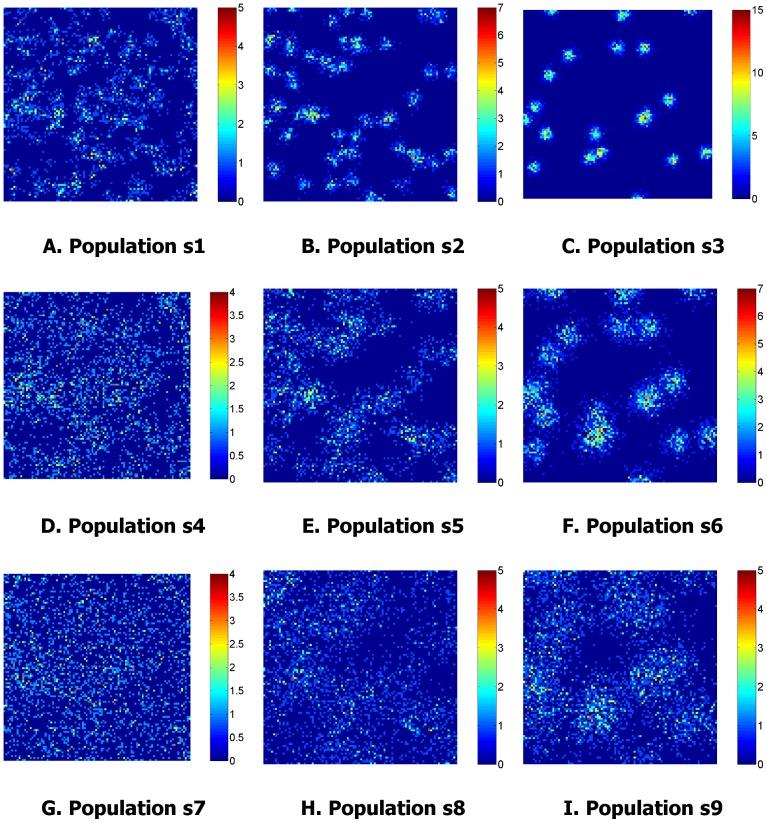
Intensity per unit quadrat for one realisation of populations s1…s9. The point process is converted into the intensity map by dividing the survey into unit quadrats and counting the number of events lying within each quadrat.

The point process was then converted into an intensity map by dividing the survey area into unit quadrats and counting the number of events lying within each quadrat. One realisation of the intensity maps for each population is shown in Figure 3. These maps served as a ‘ground truth’, and represent the function that the GP attempted to model using sparse data.

In order to be consistent with the experiment carried out by Cowling, nine vertical, equally spaced transects of length 100 were used as the training data for the GP. All quadrats along each transect were used as training data, the effective area surveyed was thus 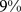 of the total study area.

The GP models were generated using a squared exponential covariance function with additive Gaussian noise. The hyperparameters for the covariance function were selected by maximising the LML.

**Figure 4:**
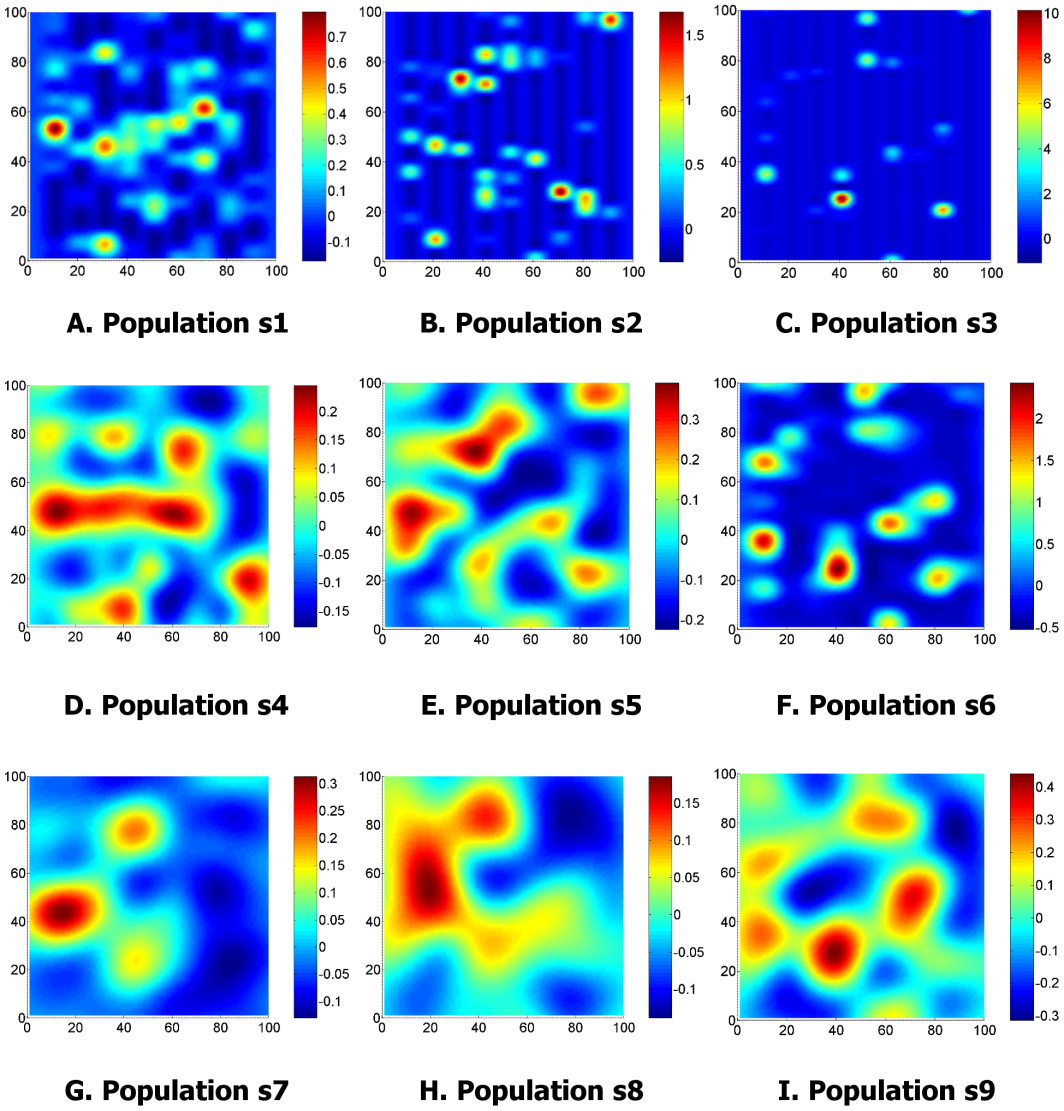
GP mean functions for one realisation of populations s1…s9. These can be considered the GP estimates of the intensity maps in the previous figure, inferred from the sparse transect data provided to the model.

**Figure 5:**
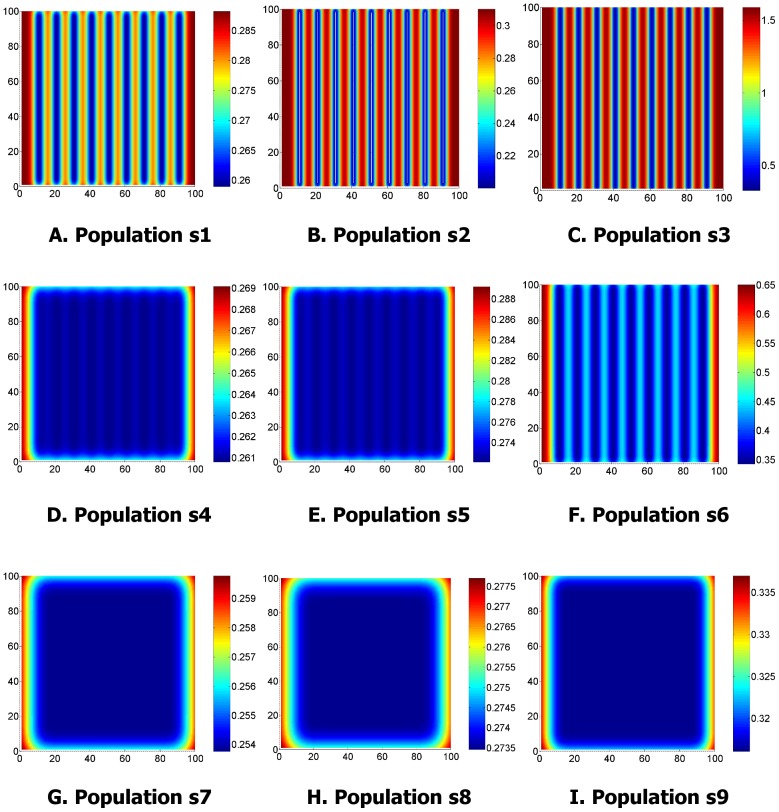
GP variance for one realisation of populations s1…s9. Note how the uncertainty increases from distance increases with distance from the vertical transects where measurements are taken.

Figures 4 and 5 show the resulting GP mean and variance for the realisation of the populations depicted in Figures 2 and 3. The variance plots show how the uncertainty increases with distance from the vertical transects. For populations s1, s2 and s3 where 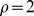 the variance increases relatively rapidly from the sample locations, as the offspring are clustered closely to the parents and inference is weak beyond the extents of a cluster. For populations s7, s8 and s9 where 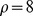, the pattern is much smoother. The variance is almost constant across the survey field, only rising at the edges where extrapolation is occurring rather than interpolation.

**Figure 6:**
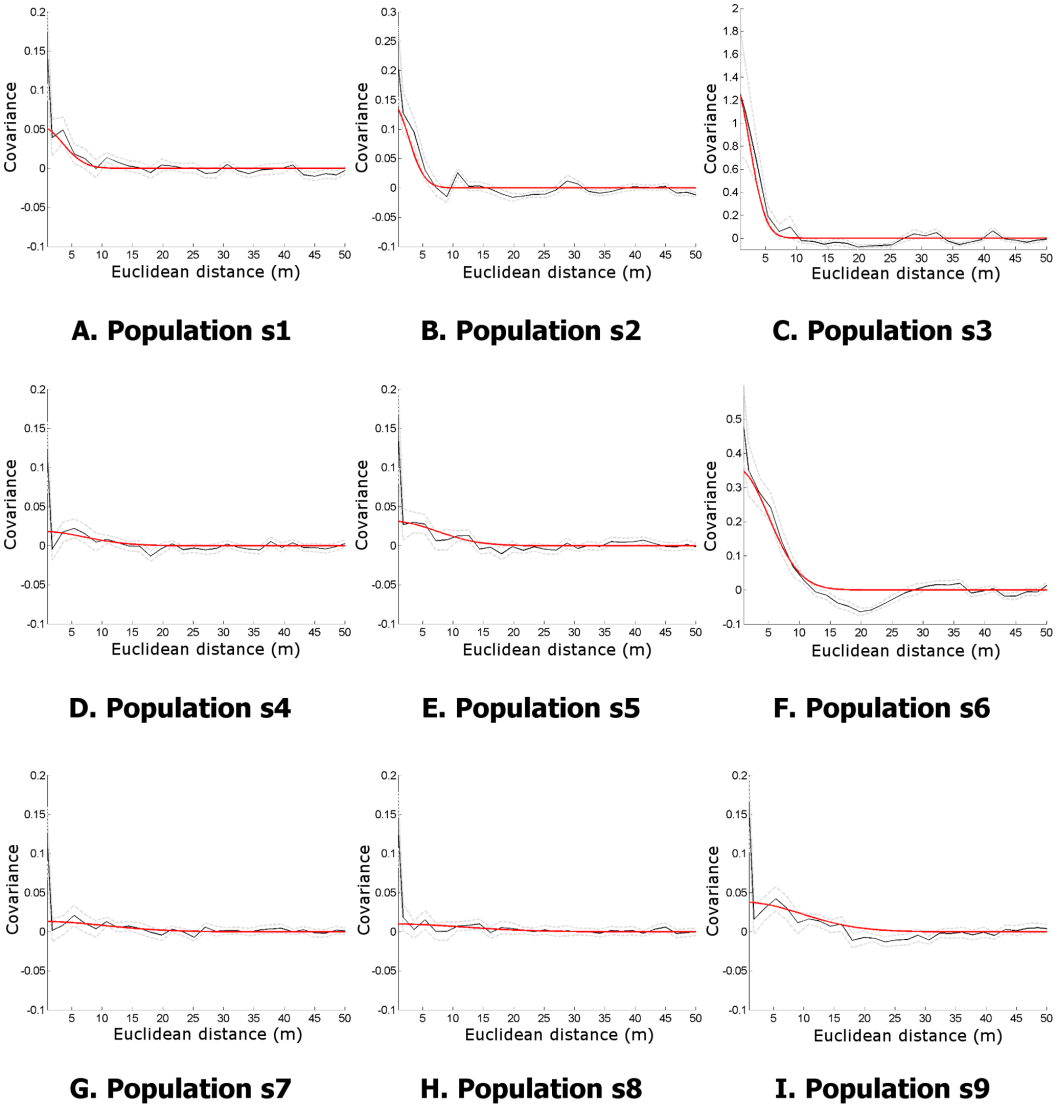
Covariance structure of GP models. The solid black line shows the mean covariance between quadrats on the true intensity map, plotted against separation (binned into unit intervals). The dotted grey lines show the standard deviation in the covariance. The solid red line shows the GP squared exponential function with the learnt hyperparameters.

For each population, Figure 6 shows the average covariance between samples plotted against their separation. The GP squared exponential covariance function with the learnt hyperparameters is superimposed in each case.

The parameters of the Neyman-Scott process were then estimated by fitting the theoretical covariance function given in Equation 22 to the optimised GP covariance function. In practice this was performed by minimising
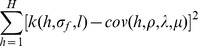


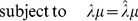
(23)where 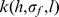 is the squared exponential covariance function given in Equation 14 evaluated for two points distance 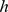 apart with hyperparameters 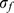 and 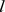; 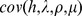 is the theoretical covariance function; 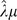 is an estimate of the mean intensity obtained by taking the mean of the training points; 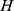 is the maximum range for the minimisation such that 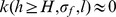.

The simulation was repeated 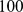 times and the means and standard deviations of the parameter estimates are given in Table 2. Corresponding results from Cowling are also reproduced as a benchmark, however a direct comparison is not entirely appropriate for two reasons. Firstly, because to our knowledge these results were not recomputed and republished once an error in Cowlings method was corrected in [15]. Secondly, Cowling's method assumes that an observer is travelling along the one-dimensional transect line, and events become exponentially more difficult to detect as perpendicular distance from the transect increases. Hence although the effective area surveyed was 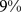 of the total study area (as in this experiment) the detection function ensures that only some fraction of events are detected. This contrasts with the GP estimator, where it is assumed that all events within a quadrat are detected.

**Table 2:** Neyman-Scott Parameter Estimates.

Population	Parameter	Value	GP Estimator	Cowling Estimator
s1	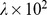	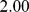	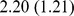	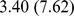
	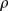	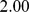	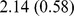	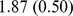
	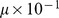	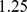	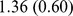	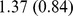
s2	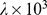	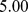	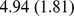	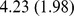
	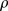	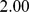	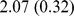	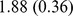
	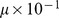	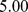	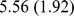	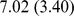
s3	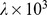	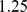	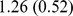	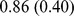
	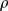	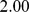	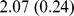	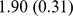
	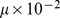	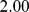	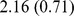	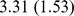
s4	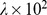	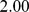	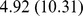	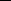
	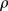	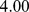	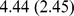	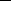
	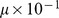	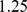	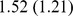	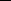
s5	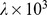	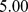	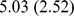	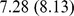
	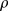	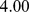	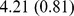	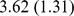
	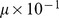	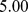	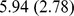	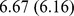
s6	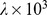	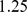	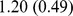	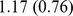
	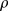	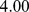	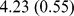	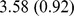
	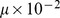	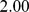	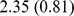	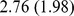
s7	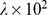	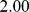	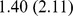	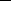
	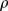	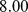	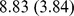	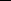
	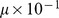	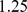	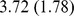	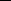
s8	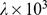	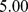	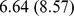	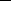
	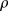	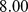	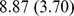	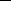
	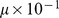	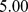	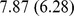	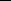
s9	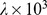	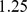	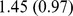	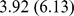
	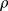	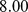	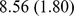	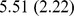
	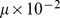	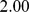	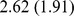	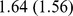

Mean and standard deviation (in parentheses) of GP parameter estimates from 100 simulated runs.

Table 2 shows that in the strongly clustered populations s2, s3 and s6 the clustering parameters can be estimated much more reliably than in the weakly clustered populations s4, s7 and s8. In the weakly clustered populations Cowling's method fails to detect any noticeable clustering, and so parameter estimation was not attempted. The GP estimator typically detects the weak clustering and tends to fit a smooth function with a very large length scale (as shown in Figures 4(d),(g) and (h)). However, occasionally (i.e. 9 runs out of 100 for population s7) the GP fails to detect the clustering, and instead overfits a spiky function with a very short length scale. Despite these outliers, on average the GP gives reasonable, if highly variable estimates for the weakly clustered populations.

In all cases the GP estimator tends to overestimate 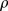 and 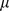. All the parameter estimates compare favourably with the K-function method of Cowling, however it must be remembered that the two experiments involve a different form of information loss: Cowling's method assumes that not all events are detected, and the GP method aggregates all event locations into a single quadrat count and so some high resolution position data is disregarded.

### Alternative Sampling Designs

**Figure 7:**
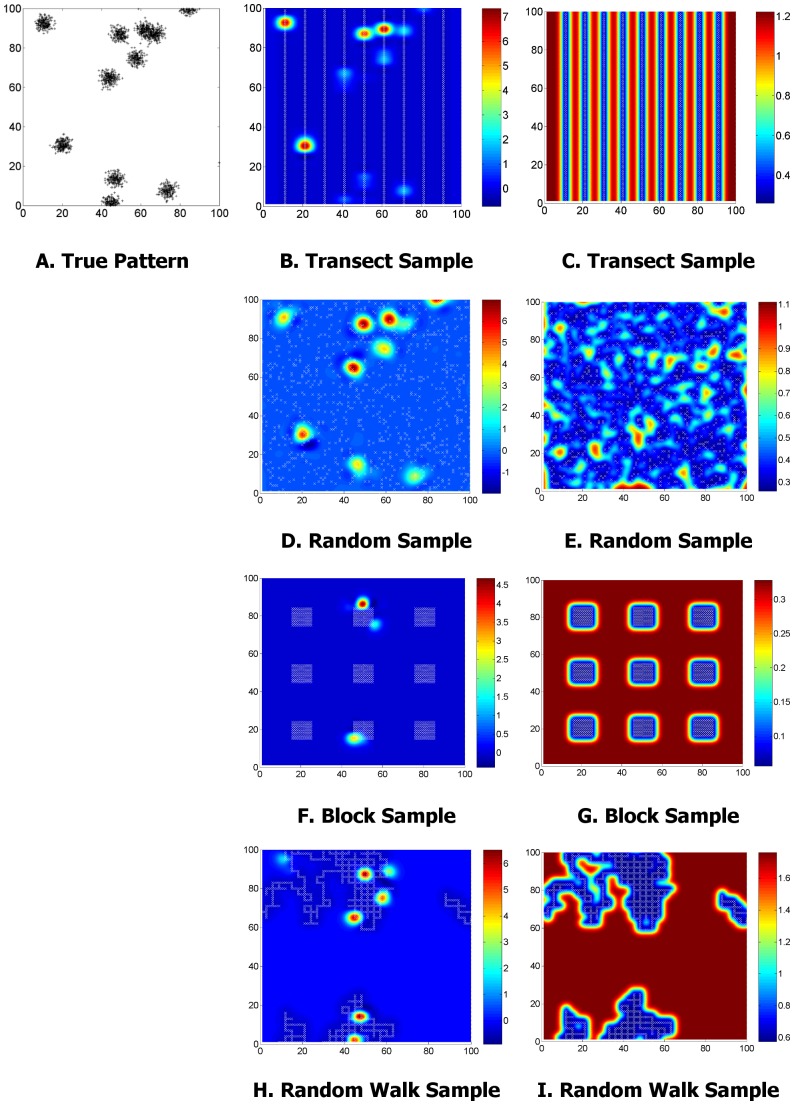
Results of run 1 for GP modelling of population s3, with various sampling designs. Left column: the actual population. Centre column: GP mean estimate of the intensity function with different sampling strategies. Sample locations are marked with a white x. Right column: Corresponding GP variance.

The previous section demonstrated that a GP can be used to estimate clustering parameters with an accuracy that compares favourably with existing methods. However the real advantage of this method is its resilience to arbitrary sampling designs. The experiment was repeated for the most clustered population s3 using three different sampling strategies: samples at uniformly distributed random points; ‘block’ sampling (nine 10×10 grids of samples with equal spacing between each grid, see figure 7); and a random walk starting at a random location. Figure 7 shows the GP mean and variance, along with the sample locations for the first run of this experiment. The transect and block patterns were identical for each run, but the random point and walk patterns were different for each run.

All of the new surveys were designed to give the same 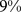 coverage of the survey field. The results are shown in Table 3, with the corresponding results from Table 2 reproduced for comparison.

**Table 3:** Parameter estimates with alternative sampling designs.

Sampling Strategy	Parameter	Value	GP Estimator
Transects	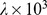	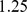	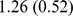
	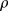	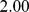	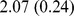
	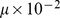	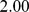	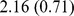
Random Points	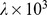	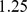	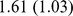
	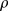	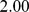	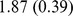
	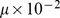	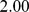	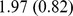
Blocks	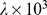	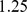	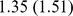
	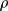	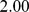	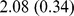
	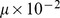	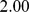	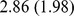
Random Walk	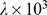	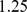	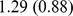
	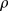	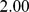	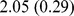
	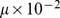	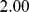	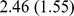

Mean and standard deviation (in parentheses) of GP parameter estimates from 100 simulated runs, for population s3 with different sampling strategies.

For this population, on balance the transect sample seems to be the most consistent estimator of the parameters. We postulate that this is because transect sampling gives reasonably even coverage of the survey field, plus contiguous samples. The former is required for the model to detect the intensity of the clusters (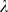), while the latter helps the model fit to the distribution of the clusters themselves (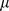 and 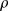). Random point sampling provides the best coverage of the survey field and so the GP mean is likely to be the most representative of the true pattern. This is evident from Figure 7(d) where almost all of the clusters are detected and modelled. However, the random point sample provides the worst estimate of 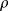. The other sampling designs all contain contiguous samples, which would be preferred in this case because the covariance is only detectable with separation between quadrats of up to approximately 5 units. Figure 7(f) provides an indication of why the block sample estimates have a very high variance; the GP mean will be unrepresentative of the underlying pattern on many of the runs if little or no clusters fall within the blocks. However when a cluster does fall within one of the blocks it will be modelled precisely, hence the good estimate of 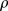. These results show that while some survey designs are better than others, the GP estimator is applicable to any survey design and provides a similar estimate of the parameters in all cases.

**Figure 8:**
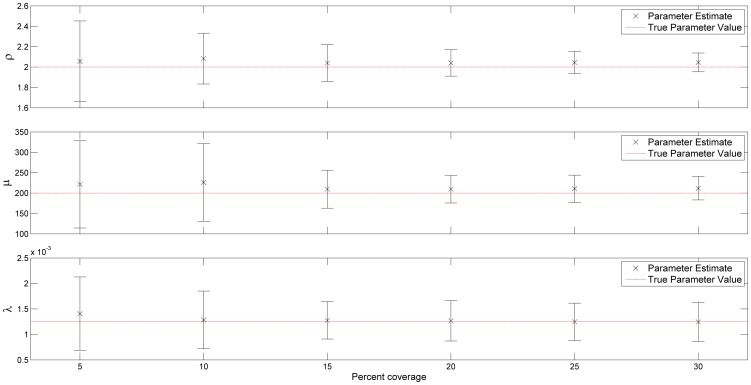
Sensitivity of method to sample size. Summarised results of 100 runs of GP modelling of population s3, for 6 transect sample designs with varying numbers of evenly spaced transects. The error bars show one standard deviation from the mean estimate.

To investigate how sensitive the method is to sample coverage, the transect sample experiment was repeated with a varying number of transect lines. As before, the experiment was run with 100 realisations of population s3 with equally spaced transect lines covering 5% to 30% of the survey area in steps of 5%. The resulting parameter estimates are shown in Figure 8. From this figure, the diminishing returns on increasing sample size are apparent. The variability in the estimate decreases significantly from 5% to 20% coverage, but increasing coverage further has little noticeable effect.

In these experiments unit quadrat size is arbitrarily used, but in practice choice of quadrat size relative to the survey area would be another variable to consider when designing the sample. Too small a quadrat size will result in a very sparse intensity map, which may result in a GP model that is ‘overfitted’ to a small number of detected events. If the quadrat size is too large, multiple clusters of events will be aggregated within a single quadrat, and this loss of resolution may result in a GP model that is ‘underfitted’, with individual clusters smoothed out. In both these extremes one would expect the estimator to perform poorly. In these experiments, the unit quadrat size is less than the standard deviation of the clusters (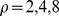) but of a similar order of magnitude; in practice we would recommend a similar approach if some prior knowledge of the underlying process is available.

## Conclusions

We have shown that a GP can provide a good model for a stationary, isotropic cluster process such as the Neyman-Scott model. As a GP is completely defined by its mean and covariance function, these provide a good proxy for the parameters of a cluster process. The GP model can be used to estimate these parameters, with a precision and accuracy which compares well with other methods within the literature. The main advantage to the GP method is its resilience to arbitrary sampling design.
